# Relationship between Night Eating Syndrome and Self-esteem: A Cross-sectional Population-based Study in Karachi, Pakistan

**DOI:** 10.7759/cureus.5540

**Published:** 2019-08-31

**Authors:** Simran Batra, Rohan Kumar Ochani, Zahid Ali Memon, Asim Shaikh, Nazuk E Qureshi, Sameer Bhimani, Muhammad Khalid Abbasi, Arsala Farhan, Suha S Qureshi, Kheenpal Das

**Affiliations:** 1 Internal Medicine, Dow Medical College, Dow University of Health Sciences, Karachi, PAK; 2 Internal Medicine, Dow University of Health Sciences, Karachi, PAK; 3 Surgery, Civil Hospital Karachi, Dow University of Health Sciences, Karachi, PAK; 4 Internal Medicine, Jinnah Sindh Medical University, Karachi, PAK; 5 Internal Medicine, Ziauddin Medical College, Ziauddin University, Karachi, PAK; 6 Internal Medicine, Ziauddin University, Karachi, PAK; 7 Psychiatry, Civil Hospital Karachi, Dow University of Health Sciences, Karachi, PAK

**Keywords:** night eating syndrome, self-esteem, eating disorders

## Abstract

Background

The amount of literature shedding light upon eating disorders in developing countries, such as Pakistan, is scarce. This is partially because talking about such matters is considered taboo in the general population. Night Eating Syndrome’s (NES) link with depression and obesity has been established; however, presently, no study has been conducted which solely focuses on NES’s correlation with self-esteem. Therefore, to bridge this knowledge gap, we conducted this study to assess the prevalence of NES in Karachi and its association with self-esteem.

Methods

We conducted a cross-sectional study in August 2018 using convenience sampling in 395 individuals, out of which 197 belonged to the age group 18-24 and 198 to 25-30. The participants were interviewed for their gender, body mass index (BMI), and their level of education. The participants were asked to complete a structured, standardized questionnaire assessment, which comprised of questions from the Night Eating Questionnaire (NEQ) and Rosenberg Self-Esteem scale (RSE). The eating habits of the participants and the level of self-esteem were assessed using four- and five-point Likert scales. Kruskal-Wallis and chi-square tests were used as the primary statistical tests.

Results

Out of the 395 respondents, more than half of the respondents were females (n = 235/395, 59.5%). About one-fourth (n = 92/395, 23.3%) of the participants had a BMI of greater than 25.0 kg/m^2^. More than one-third of the underweight (n = 20/55, 36.4%) and overweight population (n = 33/92, 35.9%) had low self-esteem, while more than one-fourth (n = 25/92, 27.2%) of the overweight participants fulfilled the criteria of NES. The final outcomes showed that 14.4% of the participants had NES, and 4.6% of the participants had low self-esteem.

Conclusion

Our results pointed out to a significant relationship between NES and self-esteem. Furthermore, NES and self-esteem also had a significant association with age, gender, and BMI. Additionally, awareness regarding eating and mental disorders should be done in countries like Pakistan, where talking in regard to such matters is considered taboo. Given the various factors that further strengthen the positive relationship between NES and low self-esteem, these factors can be the targets on which the treatment can be focused.

## Introduction

Over the past few decades, researchers have shown a growing interest in eating disorders and their relationships with psychological distress. One of these disorders, first recognized by Stunkard et al., is Night Eating Syndrome (NES). NES is a condition characterized by nocturnal hyperphagia, insomnia and morning anorexia [1]. American Psychiatric Association (APA) has recognized NES in the fifth edition of the Diagnostic and Statistical Manual of Mental Disorders (DSM-5), under Feeding or Eating Disorders Not Elsewhere Classified (2013) [2].

Literature has shown that NES is prevalent in only 1.5% of the general population [3]; however, it is prevalent in 10.1% of the class II-III obese adults, thus establishing a link between obesity and NES [4]. Other known relations are with anxiety, depression, failure to lose weight and impulsivity. Furthermore, it has been observed that NES symptoms are more likely to occur in females than in males [5]. Not surprisingly, owing to higher levels of stress, 20 out of 210 (9.52%) college students fulfill the diagnostic criteria of NES, which is around six to nine times higher than the general population [6].

Several studies in the past discuss NES’s association with depression. For instance, a study done by de Zwaan et al. concluded that more than half (n = 59/106, 56%) of the NES patients met the diagnostic criteria for lifetime major depressive disorder [7]. However, minimal studies are focusing solely on low self-esteem as a result of NES.

In Pakistan, not much light is shed upon eating disorders, and issues relating to mental health are considered a taboo; hence, there has been a noticeable rise in the number of people suffering from such problems. A study conducted in 2014 showed that almost two-thirds (n = 736/1134, 64.9%) of the female students in Islamabad scored a two or higher on the Sick, Control, One, Fat, Food (SCOFF) scale which was used to predict the possible incidence of anorexia nervosa or bulimia nervosa in 16-20-year-olds [8].

One of the principal reasons for the conduction of the study was to check for the prevalence of NES in the population of Karachi; the most populous urban city of Pakistan. We hypothesized that NES could lead to low self-esteem and also checked for any significant associations of NES with demographics such as age, gender, and education. However, no study has been conducted in Pakistan focusing on NES, and its sole association with self-esteem makes it the first of its kind in the world. To fill this knowledge gap, we carried out a study to find out the interdependence between NES and self-esteem level.

## Materials and methods

This cross-sectional study was carried out in Karachi, Pakistan, during August 2018. The sample size computed using OpenEpi was 384, after taking a confidence interval of 95% and a frequency outcome factor of 50%. A total number of 420 participants were interviewed using convenient sampling, out of which, 20 participants refused to fill the questionnaire, and a further five participants did not fill out the questionnaire completely. Therefore, the cooperation rate was 94.1%. We ensured complete anonymity, and written consent was taken.

The sample population consisted of young males and females between the ages of 18 and 30. This study was conducted in areas of high socio-economic standards within the city, only including people with an educated background. Two separate age groups were made according to the inclusion criteria of the study. The first age group comprised of people between 18-24 years while the other consisted of people between 25-30 years. People who were uneducated, did not live in the areas of high socio-economic standards, and were not between 18-30 years old were excluded from the study.

Two groups of two interviewers collected the data using a standard protocol with all subjects. All interviewers were ordered to wear identical lab coats, offer prepared explanations for questions, not to engage in mundane conversations and offer the same amount of time to each person. Incompletely filled questionnaires were discarded, and no imputation methods were used to maintain an accurate representation of the views of the sample population.

A structured, standardized questionnaire was used to collect the data. The questionnaire was divided into three sections, consisting of 27 questions. The first section was to establish demographical details while the second section was based on the Night Eating Diagnostic Questionnaire (NEQ). The NEQ was further classified by Allison et al. into four sub-scales, namely, morning anorexia, evening hyperphagia, mood, and sleeping patterns, and nocturnal ingestions. Moreover, two diagnostic threshold scores were put forward by the author, 25 and 30, which indicated high sensitivity and high specificity, respectively [9]. Items 13, 15, 16, and 17 were excluded from the survey as they rule out parasomnia, nocturnal sleep-related eating disorder, used as a descriptor of the course of the symptom and confirm the presence of distress or impairment if NES is present, respectively. The third section was based on the Rosenberg Self-Esteem Scale (RSE) [10]. According to the scale, a score between 15-25 indicates normal levels while that below 15 indicates low self-esteem [11].

Data were entered and analyzed using Statistical Package for the Social Sciences (SPSS) v. 25.0 (IBM Corp., Armonk, New York). Normality was assessed by the Shapiro Wilk test. Descriptive statistics were used to report frequencies and proportions for categorical responses. Chi-Square test was used to check for the disparity between categorical variables and final NEQ results and between categorical variables and final self-esteem level results. In the case of ordinal data, the Kruskal-Wallis test was used. A p-value of less than 0.05 was considered significant in all cases.

## Results

The final sample population consisted of 395 participants out of which 197 belonged to the age group between 18-24 (49.9%) and 198 belonged to the age group between 25-30 (50.1%). Out of the 395 participants included in the analysis, the majority were females (n = 235/395, 59.5%). Table 1 shows the demographic characteristics of the participants. It also shows the total number of participants having NES and self-esteem level. About one-fourth of the participants were overweight which included participants whose body mass index (BMI) was between 25.1-29.9 kg/m^2^ (n = 92/395, 23.3%) and who were obese with a BMI of more than 30 kg/m^2^ (n = 4/395, 1.00%). In total, 14.4% (n = 57/395) were found to have NES, and a minor proportion of participants (n = 18/395, 4.60%) were found to have a lesser than normal self-esteem.

**Table 1:** Socio-demographic variables of all the participants NES: Night Eating Syndrome; BMI: Body Mass Index.

Basic characteristics	n		Frequency (n)	Percentage (%)
Gender	395	Male	160	40.5
		Female	235	59.5
Age (years)	395	18–24	197	49.9
		25–30	198	51.1
BMI (kg/m^2^)	395	Less than 18.5	55	13.9
		18.5–25	244	61.8
		25.1–29.9	92	23.3
		More than 30	4	1.00
Level of education	395	Undergraduate	0	0
		Matriculation/O levels	4	1
		Intermediate/A levels	118	29.9
		Graduate	200	50.6
		Higher	73	18.5
Final outcomes				
NES	395	Affected	57	14.4
		Not Affected	338	85.6
Self-Esteem level	395	Low	18	4.6
		Normal	104	26.3
		High	273	69.1

Table 2 shows the correlation of NES with gender, age, BMI, level of education and self-esteem level. A significant correlation was seen between NES and gender (p = 0.001), between NES and age (p = 0.001), between NES and BMI (p = 0.003) and between NES and self-esteem level (p = <0.001). Self-esteem level showed a significant relationship with gender (p = 0.008) and BMI (p = 0.010) as shown in Table 3.

**Table 2:** Basic characteristics and self-esteem correlated with Night Eating Syndrome (NES) BMI: Body Mass Index

Characteristics		n	NES			
			Affected (%)	Not Affected (%)	n	p-value
Gender	Male	160	31 (19.4)	129 (80.6)	395	0.001
	Female	235	26 (11.1)	209 (88.9)		
Age (years)	18–24	197	17 (9.6)	180 (90.4)	395	0.001
	25–30	198	40 (20.2)	158 (79.8)		
BMI (kg/m^2^)	Less than 18.5	55	5 (1.8)	50 (98.2)	395	0.003
	18.6–25.5	244	27 (11.1)	217 (88.9)		
	25.6–29.9	92	25 (27.2)	67 (72.8)		
	More than 30	4	0 (0.0)	4 (100)		
Level of education	Matriculation/O levels	4	3 (75.0)	1 (25.0)	395	0.407
	Intermediate/A levels	118	15 (12.7)	103 (87.3)		
	Graduate	200	27 (13.5)	173 (86.5)		
	Higher	73	14 (19.2)	59 (80.8)		
Self-Esteem Level	Low	104	28 (26.9)	76 (73.1)	395	<0.001
	Normal	273	29 (10.6)	244 (89.4)		
	High	18	0 (0.0)	18 (100)		

**Table 3:** Basic characteristics correlated with level of self-esteem BMI: Body Mass Index

Basic characteristics		N	Level of self-esteem			n	p-value
			Low (%)	Normal (%)	High (%)		
Gender	Male	160	35 (21.9)	112 (70.0)	13 (8.1)	395	0.008
	Female	235	69 (29.4)	161 (68.5)	5 (2.1)		
Age (years)	18–24	197	51 (25.9)	139 (70.6)	7 (3.5)	395	0.602
	25–30	198	53 (26.8)	134 (67.7)	11 (5.5)		
BMI (kg/m^2^)	Less than 18.5	55	20 (36.4)	32 (58.2)	3 (5.4)	395	0.010
	18.5–25.0	244	51 (20.9)	181 (74.2)	12 (4.9)		
	25.1–29.9	92	33 (35.9)	57 (62.0)	2 (2.1)		
	30 or above	4	0 (0)	3 (75.0)	1 (25.0)		
Level of education	Matriculation/O levels	4	2 (50.0)	2 (50.0)	0 (0)	395	0.704
	Intermediate/A levels	118	34 (28.8)	81 (68.6)	3 (2.6)		
	Graduate	200	50 (25.0)	139 (69.5)	11 (5.5)		
	Higher	73	18 (24.7)	51 (69.9)	4 (5.4)		

Table 4 shows the frequency, most popular response of every individual question and association between individual NEQ items and gender, age, BMI, level of education and self-esteem level. According to the results, a significant relationship was seen between nine NEQ items and self-esteem level. Additionally, 29.0% (n = 115/395) of the participants agreed to be moderately hungry in the morning, while more than two-fifths (n = 165/395, 41.7%) said to have their first meal before 9 am. A quarter (n = 108/395, 27.3%) of the participants somewhat had cravings after supper and before bedtime, but a majority (n = 125/395, 31.8%) had only a little control over eating during that time. Half of the respondents (n = 198/395, 50.1%) consumed less than 25% of their daily food intake after suppertime.

**Table 4:** Individual Night Eating Questionnaire (NEQ) items, most popular response and correlation with basic characteristics and Night Eating Syndrome (NES)

Items	No. of responses (n)	Percentage (%)	Most popular response (%)	p-value				
				Gender	Age (years)	BMI (kg/m^2^)	Level of education	Self-esteem level
1. How hungry are you usually in the morning?	395	100	Moderately (29.0)	0.570	0.022	0.850	0.024	0.005
2. When do you usually eat for the first time?	395	100	Before 9 am (41.7)	0.011	0.561	0.323	0.689	0.131
3. Do you have cravings or urges to eat snacks after supper, but before bedtime?	395	100	Somewhat (27.3)	0.201	0.778	0.100	0.972	0.033
4. How much control do you have over your eating between supper and bedtime?	395	100	Some (31.8)	0.363	0.013	0.037	0.110	0.954
5. How much of your daily food intake do you consume after suppertime?	395	100	1-25% (Up to a quarter) (50.0)	0.079	0.027	0.459	0.459	0.524
6. Are you currently feeling blue or down in the dumps?	395	100	Not at all (31.8)	<0.001	0.500	0.003	0.072	<0.001
7. When you are feeling blue, is your mood lower in the:	395	100	Late evening/Nighttime (34.5)	0.693	0.463	0.103	0.167	0.017
8. How often do you have trouble getting to sleep?	395	100	Sometimes (50.4)	0.983	0.472	0.099	0.140	<0.001
9. Other than only to use the bathroom, how often do you get up at least one in the middle of the night?	395	100	Never (41.0)	0.536	0.138	0.118	0.947	0.001
10. Do you have cravings or urges to eat snacks when you wake up at night?	245	62.0	Not at all (52.1)	0.669	0.001	0.039	0.298	0.033
11. Do you need to eat to get back to sleep when you wake up at night?	245	62.0	Not at all (68.5)	0.686	<0.001	0.114	0.001	0.038
12. When you get up in the middle of the night, how often do you snack?	245	62.0	Never (54.2)	0.625	<0.001	0.015	0.052	0.021
13. How much control do you have over your eating while you are up at night?	245	62.0	Complete (27.7%)	0.698	0.014	0.055	0.201	0.597

Moreover, two-thirds of the respondents stated to be currently feeling blue (n = 269/395, 68.2%) and a majority (n = 136/395, 34.5%) said to be feeling it during late evening and nighttime. Half of the participants (n = 199/395, 50.4%) had troubles getting sleep sometime, and about two-thirds (n = 233/395, 59%) woke up at least once in the middle of the night besides to use the restroom. About half (n = 117/245, 47.9%) of such participants had cravings to eat snacks when they wake up, but two-thirds (n = 168/245, 68.5%) did not need to eat in order to go back to sleep. More than half (n = 133/245, 54.2%) never had a snack when they woke up in the middle of the night and a quarter had (n = 68/245, 27.7%) complete control over their eating during the night.

Apart from this, seven NEQ items proved to have a significant relationship with age while only four had a significant relationship with BMI. Gender was found out to have a significant relationship with items two (p = 0.001) and six (p = <0.001), while level of education had a significant relationship with items one (p = 0.024) and 11 (p = 0.001).

Similarly, Table 5 shows the frequency, most popular response and the association between individual RSE questionnaire items and gender, age, level of education and NES. According to the results, all items of RSE showed a significant relationship with NES, gender, and age. A majority (n = 275/395, 69.6%) of the participants agreed to be overall satisfied with themselves, while more than half (n = 224/395, 56.7%) agreed to think they are no good at all. A vast majority (n = 350/395, 88.7%) felt that they had several good qualities and 83.3% (n = 329/395) believed that they could do things as well as most other people. Two-fifths of the respondents (n = 167/395, 42.3%) felt that they do not have much to be proud of, and more than half (n = 226/395, 57.2%) felt useless at times.

**Table 5:** Individual Rosenberg Self-Esteem Scale (RSE) items, most popular response and correlation with basic characteristics and Night Eating Syndrome (NES)

Items	No. of responses (n)	Percentage (%)	Most popular response (%)	p-value				
				Gender	Age (years)	BMI (kg/m^2^)	Level of education	NES
14. Overall, I am satisfied with myself.	395	100	Agree (55.8)	<0.001	0.001	<0.001	0.165	<0.001
15. At times I think I am no good at all.	395	100	Agree (43.7)	<0.001	<0.001	0.337	0.370	<0.001
16. I feel that I have several good qualities.	395	100	Agree (64.8)	<0.001	0.002	0.392	0.278	<0.001
17. I can do things as well as most other people.	395	100	Agree (55.8)	<0.001	<0.001	0.945	0.260	<0.001
18. I feel I do not have much to be proud of.	395	100	Disagree (42.9)	<0.001	<0.001	0.014	0.661	<0.001
19. I certainly feel useless at times.	395	100	Agree (45.2)	<0.001	<0.001	0.017	0.254	<0.001
20. I feel that I am a person of worth, at least on an equal plane with others.	395	100	Agree (65.2)	0.003	0.001	0.074	0715	<0.001
21. I wish I could have more respect for myself.	395	100	Agree (41.4)	<0.001	<0.001	0.026	0.556	0.001
22. All in all, I am inclined to feel that I am a failure.	395	100	Disagree (47.6)	0.001	<0.001	0.018	0.324	<0.001
23. I take a positive attitude towards myself.	395	100	Agree (51.4)	<0.001	<0.001	0.052	0.012	<0.001

Furthermore, a considerable majority (n = 331/395, 83.8%) felt that they are worthy as a person or at least on an equal plane as others, but contrary to that two-thirds of the participants (n = 260/395, 65.8%) agreed that they wish they had more respect for themselves. Finally, a quarter agreed to have inclined themselves to feel that they are a failure, while a majority (n = 306/395, 77.4%) took a positive attitude towards themselves.

Lastly, items one (p = <0.001), 18 (p = 0.014), 19 (p = 0.017) and 22 (p = 0.018) were found out to have a significant relationship with BMI. While on the other hand, only item 23 (p = 0.012) was found out to have a significant relationship with level of education.

## Discussion

Our primary finding was the presence of a significant relationship between individuals suffering from NES and low self-esteem. This finding is congruent to previous studies, which all found a similar relationship [12,13]. This association can be better explained by a multi-faceted approach. Firstly, the link between NES and obesity has been clearly established [14], which when coupled with the fact that overweight individuals suffering from NES also find it much harder to lose weight, as compared to overweight individuals who do not suffer from this condition, suggests that a proportion of the self-esteem issues are derived from body image issues [15]. In addition, NES represents eating at different times which has been proven to affect the circadian rhythm profiles and to influence various hormone levels of individuals suffering from NES [16]. These fluctuations have been linked to adverse effects on sleep, appetite, and mood, as well as to the initiation of anxiety disorders, which further justifies the low self-esteem observed in these individuals [17].

Furthermore, a history of depression, anxiety, and substance abuse are common in individuals suffering from NES [18]. These factors alone, without the presence of NES, are also linked with extremely low self-esteem, therefore it is essential to recognize that the cause of low self-esteem in these individuals could be due to the presence of different combinations of the previously mentioned factors in addition to the presence of NES [19,20]. Also, recently, a possible genetic link to NES has been proposed. It is believed that the PER1 gene could be the cause of why obese individuals develop NES [21].

We found a statistically significant association between the presence of NES and the male gender. This is contrary to previous studies which suggest that there is no difference amongst genders with respect to NES [22,23]. This finding can be explained by the fact that the presence of NES is predicted by a number of psychosocial behaviors such as skipping meals and night snacking which, as a study found, can be more prevalent in males [24].

We also found a significant relationship between adults aged 25-30 having NES as opposed to younger individuals. This finding is congruous to a previous study, which suggests that NES is more common in older adults because consecutive night eating can take a long time to cause a large increase in the body mass of an individual and hence, there is a lack of reporting of NES in very young individuals [25]. Furthermore, a study found that the age at which majority of individuals reported first becoming obese was 26 [22]. This supports our finding that NES is more prevalent in the 25-30 age group as individuals become more susceptible to obesity and hence, NES, during this age.

Additionally, we found that the underweight (<18.5 kg/m^2^) and overweight population (25.1-29.9 kg/m^2^) was associated with the prevalence of NES and low self-esteem, especially owing to the social perception regarding body image [26]. This finding has been reported multiple times [12,27,28]. It can be explained by the fact that obesity is a direct cause of NES [28], and low self-esteem [27]. Furthermore, the presence of body dissatisfaction and low self-esteem has shown a strong association in the past, which supports our finding correlating underweight individuals and NES, as underweight individuals, in order to gain weight, may have altered their eating habits and circadian rhythms which eventually may have led to NES [29]. Documented evidence shows that NES has been linked to the previous existence of eating disorders, and eating disorders are partially responsible in gaining weight therefore, a cycle of unhealthy dietary practices leads to increased weight and, eventually, establish NES [30].

Our study had some limitations which need to be considered. Firstly, considering that talking about psychiatric disorders is considered a taboo in our society, our participants may have chosen a socially acceptable response rather than giving an honest answer, given the method being self-reporting. Secondly, convenience sampling was used to reach the target population; therefore, the results of the study may have received a degree of bias. Finally, our targeted population was from only one city, Karachi, therefore, the results of this study cannot be generalized to the broader population; hence, more studies need to be conducted across the country to fulfill the need of the generalizability of the results.

## Conclusions

The findings of this study conclude that there is a strong association between NES and low self-esteem levels. Additionally, an abnormal BMI showed a positive correlation with NES and lower self-esteem, which calls for the need to counsel patients regarding their regular dietary intake. Considering the gravity of this topic, there is an urgent need to create awareness regarding eating disorders using different strategies and platforms. Efforts by general practitioners should likewise be made to educate people that a discussion about eating and mental disorders must not be considered taboo.
